# Dioxin Exposure and Cardiovascular Disease: An Analysis of Association

**Published:** 2008-11

**Authors:** Valerie J. Brown

Dioxins have long been known as highly toxic compounds, having been implicated in cancer, immune system disorders, endocrine disruption, and birth defects. Animal and *in vitro* studies have also suggested a role for dioxins in heart disease. Now a systematic review of epidemiologic studies has found an association between dioxin exposure and death from cardiovascular diseases, particularly ischemic heart disease (reduced blood supply to the heart) **[*EHP* 116:1443–1448; Humblet et al.]**.

The authors reviewed all English-language studies on dioxin exposure and death from cardiovascular disease reported in PubMed as of December 2007. They used a broad definition of “dioxins” that included polychlorinated dibenzofurans (PCDFs), polychlorinated biphenyls (PCBs) with dioxin-like effects, and polychlorinated dibenzo-*p*-dioxins (PCDDs). However, they excluded studies that focused primarily on PCBs because results pertaining to the non–dioxin-like members of this family would muddy the interpretation of dioxins’ cardiovascular effects. Likewise, studies of occupational exposure in the leather processing and flavor/fragrance industries were excluded because workers in these industries are exposed to cardiotoxic methylmercury, arsenic, and xylene along with dioxin.

The remaining studies included 12 dioxin-exposed cohorts, one of which was a large multicenter cohort study; 10 of these cohorts involved military or occupational dioxin exposure, and 2 involved environmental exposure of the general public. The cohorts included Vietnam veterans exposed to herbicides; the population of Seveso, Italy, exposed in 1976 by an industrial accident to 2,3,7,8-tetrachlorodibenzo-*p*-dioxin (TCDD), the most toxic dioxin; and a Taiwanese population exposed in 1979 to polychlorinated dibenzofurans in rice oil.

The researchers identified numerous potential limitations in the reviewed studies. For example, none of the studies adjusted for all the known cardiovascular risk factors of diet, smoking, physical activity, family history, and body mass index, so residual confounding was a concern. Furthermore, not all studies made internal comparisons between lowest-exposed and highest-exposed members of a cohort. Some compared the exposed cohorts with the general population, which may confound results because of the “healthy worker effect” (that is, workers as a group are typically healthier than the general population, which includes those who are too ill to work). There was also wide variation in the precision of exposure estimates.

Despite these potential limitations, the authors concluded that the literature as a whole presented reasonable evidence that dioxin exposure at high doses raises the risk of death from heart disease. They suggest the need for further studies that account for the limitations in the existing studies, particularly with respect to confounders, choice of comparison populations, and environmentally relevant exposure concentrations.

## Figures and Tables

**Figure:**
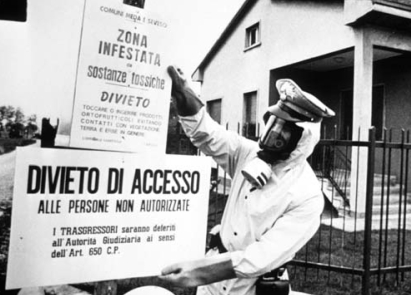
After the 1976 Seveso accident involving TCDD, an official posts a sign warning residents not to touch or eat garden produce and to avoid contact with vegetation, soil, and grass.

